# The ESCAPE Open-source Software and Service Repository

**DOI:** 10.12688/openreseurope.15692.1

**Published:** 2023-03-13

**Authors:** Thomas Vuillaume, Mohammad Al-Turany, Matthias Füßling, Tamas Gal, Enrique Garcia, Kay Graf, Gareth Hughes, Mark Kettenis, Dmytro Kresan, Jutta Schnabel, Christian Tacke, Marjolein Verkouter

**Affiliations:** 1 LAPP, Univ. Savoie Mont-Blanc, CNRS, Annecy, 74940, France; 2 GSI Helmholtz Centre for Heavy Ion Research GmbH, Darmstadt, 64291, Germany; 3 Cherenkov Telescope Array Observatory gGmbH (CTAO gGmbH), Heidelberg, 69117, Germany; 4 ECAP, Friedrich-Alexander-Universität Erlangen-Nürnberg, Erlangen, 91058, Germany; 5 IT Department, CERN, Geneva, Switzerland; 6 Joint Institute for VLBI ERIC, Dwingeloo, 7991 PD, The Netherlands

**Keywords:** EOSC, open-source, software, repository, Zenodo, ESFRI

## Abstract

**Purpose:** The purpose of the ESCAPE Open-source Software and Service Repository (OSSR) is to provide a central location for the dissemination and use of trusted open-source software in the fields of astronomy, astroparticle physics, and particle physics.

The repository allows users to easily access and download tools and services developed within the community, and to contribute their own tools and services.

**Methods:** The ESCAPE project has set up a curated repository of software that provides tools and an environment to make it easy for users to find and download the software and services that they need. The repository is regularly updated and is maintained by a curation board, ensuring that the software and services are reliable and up-to-date. The curation and onboarding process makes the OSSR a trustworthy source of software that can be used for scientific analysis. The software included in the repository must include documentation and instructions and follow a set of modern best practices in software development. Training is provided to students and researchers to help them provide high-quality scientific software following modern software development practices.

**Outcome:** The OSSR currently contains a wide range of software and services, including those for data management, data analysis, and machine learning. These tools and services are used by researchers and other users around the world. The OSSR has proven to be an effective means for disseminating and providing open-source software and services developed by the ESCAPE project partners and welcomes contributions from the entire community.

## Plain language summary

The ESCAPE Open-source Software and Service Repository (OSSR) is a place where scientists can find and download software and services they need for their work in astronomy, astroparticle physics, and particle physics. This repository is updated regularly and maintained by a group of experts to make sure that the software is reliable and open-source to maximize its reuse. The software available on the repository must come with instructions and follow good software development practices. The OSSR provides training to students, researchers, and software developers to help them create high-quality software. The OSSR includes a variety of tools and services for things like data management and analysis that are used by researchers worldwide.

## 1 Introduction

The ESCAPE project is a European H2020 project that brings together researchers and institutions from the European Strategy Forum on Research Infrastructures (ESFRI) and leading research infrastructures. Its goal is to develop new technologies and methods for dealing with the increasingly large and complex datasets that are generated in the fields of astronomy, astroparticle physics, and particle physics. The project also focuses on making these technologies and methods accessible to a wider audience, through the development of open-source software and services. The ESCAPE Open-source Software and Service Repository (OSSR) is a key component of this effort, providing a central and trusted location within the European Open Science Cloud (EOSC) for the dissemination of curated software and analysis tools. The OSSR follows a federated, community-driven approach, gathering from all researchers in the domains associated with the ESCAPE project.

In this open letter, we summarize the OSSR effort based on previously published material, and present its guidelines and views. In Section 2, we show how the OSSR has been built to provide software following the FAIR principles that are the core components of open science (based on the following OSSR publications: 1– 4). Contributions to the OSSR are curated by a committee of domain experts following clear and published policies in order to build a trusted repository. Then in Section 3, we highlight the technical components of the OSSR which is built around a Zenodo (RRID:SCR_004129) community with enriched metadata and application programming interface (API) connection (based on 2, 3, 5– 7). In Section 4, we show the importance of community building, and training software developers to write and publish FAIR software, and our efforts in this regard (based on 8– 14). Finally, we present the outcomes in Section 5 (based on 15, 16).

## 2 The OSSR goals and policy

### 2.1 Goals

The OSSR acts as a central place to share open science software and services based on FAIR principles. The objectives of the repository are to facilitate and support the continuous development, deployment, exposure, and preservation of partners’ software, tools, and services. Additionally, the repository aims to foster interoperability, research software re-use, and cross-fertilization between the members of the community. It offers an open innovation environment for open standards ( *e.g.* workflows, data formats), common practices, and shared novel software ( *e.g.* for multi-messenger and multi-probe data). The OSSR is built by and for the communities and follows an inclusive approach by federating available resources, with the goal of enabling open science and treating software as a central part of knowledge and science production. By highlighting software in research, the OSSR also emphasizes developer effort and helps to increase recognition of their contribution to science. This is possible in particular by facilitating software citation, promoting communication, collaboration, training, and recognition of developers.

### 2.2 The FAIR principles

With the implementation of the OSSR, partners from the particle physics, astroparticle and astronomy communities are able to aggregate their software in a common place. This alone is not enough to ensure that the FAIR (Findable, Accessible, Interoperable and Reusable) principles ^17^ are respected. The choice of Zenodo ^18^ as repository back-end (see Section 3.1) provides the basis for a FAIR and sustainable infrastructure ^1^. The OSSR policy furthers the adoption of the FAIR principles by imposing requirements on licensing and metadata before submissions are accepted into the OSSR (see Section 2.3). In particular, licensing ensures legal security for the user of the resources and is therefore considered an essential part of the “re-usability” requirement of FAIR resources. In addition, the usage of an extended and common metadata schema strengthens the “findability”. Guidelines for license, provenance, and metadata have been developed ^1^.

With this implementation, the OSSR follows the requirements for the FAIR principles for software ^19^. Although used as guidelines throughout the development of the OSSR service, the FAIR principles for data are not fully valid in the regime of software and services, as the SIRS report from the EOSC Architecture Working Group points out ^20^. This is predominantly because software usually follows a more complex life cycle with elements such as development and maintenance which are not covered by the FAIR principles. The OSSR addresses this by linking development platforms, the repository itself, and additional information related to the software.

### 2.3 Policy

The OSSR policy ^2^ provides the requirements, recommendations, guidelines, and user agreement to contribute to the repository and enforce its goals. The guidelines and rules of participation therefore contain as one of two obligatory clauses that the submission has a license, such that it is clear what use of the software is legal. However, the degree of openness of the license is not defined, although open licenses are recommended and various resources provided ^3^ to help to choose a suitable license.

The aim to provide high-quality software leads to a number of requirements and recommendations. In particular, a minimum amount of provenance information needs to be provided together with the software, as well as a metadata file in a common human- and machine readable format (CodeMeta, see Section 3.4.1), enabling users and services to find it, understand the status, and integrate it in their respective environments. This metadata file must be provided as part of the archived record and must include a link to the software documentation, a versioning scheme, and author contact details. Compliance with these requirements is checked during the curation process described below.

Beyond these minimal requirements, the application of software quality standards such as the use of a testing scheme is encouraged. A software checklist is provided based on the outcomes of the WOSSL workshop (see Section 4.2) which also includes external references ^2^. The choice of the Zenodo platform to host the OSSR submissions results in an implicit requirement to agree with the Zenodo terms of use ^2^ before a submission can be included in the OSSR. To ensure quality and trustability, the integration of a new record in the OSSR is submitted to a curation process, validating its compliance with the guidelines and serving as a gatekeeper to the OSSR.

### 2.4 Curation

The curation process is overseen by a board that meets regularly. This curation board initially consists of ESCAPE project members, but new members can join from the communities served by the OSSR in the future by joining the ESCAPE open collaboration. After the onboarding presentation and initial upload to Zenodo, new OSSR contributions are assigned to a board member. During curation, the items on the software checklist are checked based on the onboarding presentation, the software repository, the software documentation, and the provided metadata. The metadata itself is checked using a validator (see section 3.4.3) which signals missing required and recommended metadata. Curation is an iterative process; the curator will point the submitter of a new contribution to deficiencies and suggest improvements to meet the OSSR policy requirements. At the end of a (successful) curation process, the curator will make the Zenodo entry part of the ESCAPE community. At this point, the contribution is officially part of the OSSR.

To make sure the entries continue to meet the OSSR policy, the curation board will oversee the removal of the entries that no longer meet the requirements. Examples of reasons for removal include the software no longer being maintained, or a change to an incompatible license. Zenodo entries will never be deleted, but will no longer be part of the OSSR by being removed from the Zenodo community (see section 3.1).

## 3 The OSSR technical implementation

### 3.1 A curated community of the Zenodo repository

The core of the OSSR is implemented as a curated community, named escape2020, of the Zenodo general-purpose repository ^18^. The Zenodo repository service was chosen as it is developed as an open-source project between CERN ^3^ and the OpenAIRE project ^4^, and is hosted by CERN, a partner of the ESCAPE project ^7^. It is developed to respect the FAIR principles and is compliant with the recommendations from OpenAIRE as well as FAIR4RS ^19^. Its main features are to provide a long-term, safe and secure archive. The service is able to deal with many kinds of content and implements publication versioning, providing a unique DOI for each version, thus allowing precise citation of the used software, workflow, or dataset. Its long-term sustainability as well as machine- and human-interactionability are key aspects of the goals of the OSSR.

### 3.2 The eOSSR library

A Python3 (RRID:SCR_008394) library called eOSSR ^5, 6^ has been developed as a central tool to interact with the OSSR. The library provides an API for exchanging information with the OSSR, such as pushing new contributions or searching and retrieving existing ones. It also includes the OSSR metadata definition and requirements based on CodeMeta and means to convert and validate the metadata associated to software records. Finally, it implements OSSR policies to allow automated validations and ease of the curation process.

The eOSSR library is open-source, it has been onboarded in the OSSR, so it can be found by the community and its documentation can be found online ^5^.

### 3.3 API and GitLab to Zenodo

**Figure 1.**
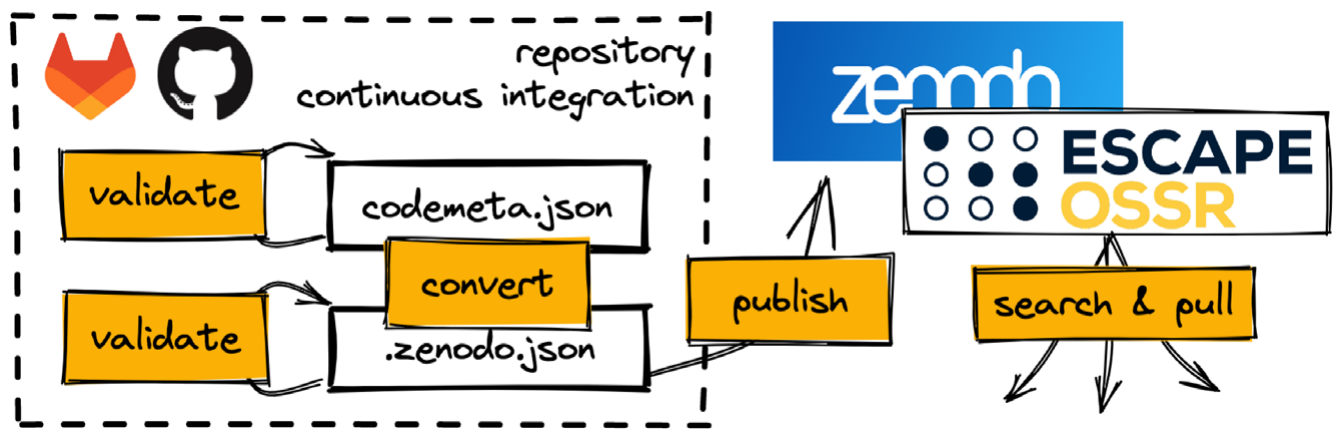
A typical software publication workflow in the Open-Source and Software Repository (OSSR) using the eOSSR library and repositories continuous integration.

The eOSSR uses Zenodo’s API to provide a set of high-level functionalities through Python functions or command-lines, such as requesting software in the OSSR through full text searches, or specific searches using recognized metadata, *e.g.* keywords or file types. The API also provides several general purpose functions to query Zenodo content such as records, communities, licenses, grants, or funders. Using Zenodo tokens, the library can be used to modify existing metadata records, or to publish new ones. This functionality eases the publication of software from GitLab ^6^ (RRID:SCR_013983) to Zenodo, offering a similar functionality to the Zenodo-GitHub integration ^7^. eOSSR provides docker containers and code snippets that can be integrated in any GitLab continuous integration (CI) to automatically publish software in Zenodo when a new version is released (see workflow in Figure 1). More information can be found in the eOSSR documentation.

### 3.4 Software Metadata

The purpose of metadata is to provide structured information about the entity it describes in order to improve its discoverability and reuse, as well as to provide information about its quality and reliability. A format like CITATION.cff ^8^, developed for software citation, is therefore not sufficient to fully describe software. The OSSR metadata is based on CodeMeta to also include links to the software code repository, documentation, and CI environment. This format meets the OSSR requirements, is extensible, and has been created specifically to describe scientific software and analysis. The choice of CodeMeta makes software version and persistent identifiers part of the metadata and helps to fulfill the FAIR4RS principles ^19^.

The FAIR4RS principles emphasise metadata for software for the Findable, Accessible, and Interoperable parts of the FAIR acronym. To make software Findable they require that the software is described by *rich* metadata, that this metadata clearly and explicitly identifies the described software and that the metadata itself is FAIR, searchable and indexable. To make software accessible and interoperable, it is required that the metadata remains accessible and can be retrieved using a standardized protocol even if the software is no longer available. The OSSR meets this requirement by making the metadata part of the Zenodo record for the software. This allows the eOSSR software library to retrieve the CodeMeta file through the Zenodo API. The OSSR metadata also answers ESFRI’s requirements to clearly identify the software providers, the affiliated packages and open-community versions while being available in the same repository. The interoperability is illustrated by the integration between the OSSR and the ESFRI Science Analysis Platform (ESAP) discussed later in this paper, see Section 5.2.

***3.4.1 CodeMeta*.** CodeMeta ^21^, is a cross-platform and common standard metadata schema, has been selected by the OSSR to describe software. It is a metadata schema based on Schema.org ^9^ (RRID:SCR_018291) and developed to describe software artifacts. It provides a standardized way to describe the software and its dependencies, as well as auxiliary information such as licenses, documentation, contact details, or development practices used to create it. It is based on the JSON-LD format, which allows the metadata to be easily embedded in the software’s source code repository or distributed as a separate file named codemeta.json.

One of the main goals of CodeMeta is to facilitate the integration of software metadata with other research and discovery tools, such as search engines, citation indexing systems, and research data repositories. By providing a common metadata standard, CodeMeta aims to improve the interoperability and reuse of software within the research community, as well as to provide a means for researchers to properly acknowledge and cite the software they use in their work.

***3.4.2 CodeMeta-Zenodo crosswalk*.** Zenodo has its own internal metadata schema for describing records such as software artifacts. CodeMeta is not yet supported natively by Zenodo, even though in the integration plan ^10^. Therefore, a converter, based on the CodeMeta official crosswalk ^11^, is provided in the eOSSR documentation to convert CodeMeta metadata into Zenodo metadata.

***3.4.3 Metadata toolbox: generator, validator, and converter*.** Based on the metadata definition, three sets of tools have been developed to ease a software provider’s life. All links and documentation to use these tools are available on the OSSR pages ^3^.

**Generator:** The official CodeMeta online generator has been adapted to generate specifically ESCAPE metadata following the CodeMeta schema.

**Converter:** A CodeMeta-Zenodo metadata converter has been implemented in the eOSSR library. It is used internally in the library during software publication so that CodeMeta can be used as the source of metadata. It can also be used directly through a command line interface, or online.

**Validator:** A validator has also been developed. It can be used as a command line tool, *e.g.* during development or in continuous integration workflows to ensure that the CodeMeta file is always compliant with the up-to-date OSSR requirements, or with its online version to help creating a valid CodeMeta file.

### 3.5 Curation and onboarding process

The onboarding process ^2^ serves two main purposes: on the one hand, the quality of the contributed software is ensured by checking the adherence to community standards, on the other hand, it offers a forum for discussion for the contributors to present their project and consider further enhancements and cooperation. The main steps in the process are:

1. registration for the onboarding process, providing contact and software references,
2. giving an onboarding presentation introducing the software,
3. adding the software to Zenodo and applying for membership in the ESCAPE OSSR community, and
4. curation of the software and acceptance in the ESCAPE OSSR Zenodo community.

In the initial setup during the ESCAPE project, the various steps were organized on standard services setup for the ESCAPE project (a Redmine platform ^12^, an Indico platform ^13^ and the GitLab (RRID:SCR_013983) IN2P3 platform ^14^). After completion of the project in January 2023, the process is reorganized on gitlab.com ^15^ ensuring centralized documentation and communication. Registration for the onboarding process (1) is managed by opening an issue, information on the contribution is collected there and, finally, the curation process is triggered (3 & 4) and managed by automatically created merge requests to a curation repository. The onboarding presentation (2), for which a template is provided, can be given at dedicated online meetings, during which presentations are recorded. The community is invited to discuss application of and cooperation for the presented software. This additional information is made available for each entry on the OSSR website ^3^.

### 3.6 Reusability

The OSSR environment and tools use technologies that allow easy adoption by other institutes or communities. The setup answers the FAIR principles and can be reused by another domain that could create its own Zenodo community and configure the external tools ( *e.g.* onboarding, eOSSR and specific metadata usage) to easily and rapidly set its own curated repository.

## 4 Community building

The OSSR mission includes the discussion and dissemination of best practices among the research community in order to improve the skills and knowledge of its researchers in building open science and therefore to grow its number of contributors. Training in the form of dedicated schools is an important part of this process, as it helps to ensure that research software developers and maintainers are able to effectively contribute. These schools provide an opportunity for participants to learn about the principles and practices of open-source software development, and to gain hands-on experience working on projects within the community. This helps to ensure that the repository is able to continue to grow and evolve over time, and that it remains a valuable resource for the ESFRIs and wider research community.

### 4.1 The ESCAPE data science schools

The ESCAPE data science schools are the continuation of the three ASTERICS-OBELICS international schools ^8– 10^ which were organized from 2017 to 2019 in Annecy (France) for PhD students, postdocs, and senior researchers working in the domain of astrophysics and astroparticle physics, as part of the ASTERICS H2020 project ^16^.

During the ESCAPE project, two schools have been organized ^11, 14^. The schools’ program includes courses on machine learning, big data, organizing coding environments, best practices in data management and collaborative research using state-of-the-art tools, continuous development and integration of software, and also low-level optimization including debugging and profiling. Python3 was chosen as the main programming language for most of the courses due to its popularity and rich ecosystem of scientific libraries and resources. The program includes theoretical and hands-on training such that participants can leave with new skills and ideas directly applicable to their own work. During the schools, participants were able to communicate with each other and the tutors *via* chat messages using Slack ^17^ to raise issues, ask questions about the courses, as well as to socialize.

The first ESCAPE data science school ^11^ was organized online due to the COVID-19 pandemic restrictions. The lectures were live-broadcasted on YouTube and their recordings are still openly available on the platform ^18^. The school took place in June 2021 with over a thousand registrants and seventeen tutors ^12, 13^ and a schedule spanned over 12 days.

In June 2022, the second ESCAPE data science school ^14^ was held, this time in person, at the original location in Annecy, France, with a restricted audience.

Following the FAIR principles, the complete material of the courses including the presentations, exercises, and their solutions were put together in public GitHub repositories ^19^, allowing a collaborative and live construction of the material and knowledge by all tutors and participants. At the end of the schools, all the content was published on the OSSR, following a similar life-cycle as software.

### 4.2 Workshop WOSSL

As part of the community building effort, a workshop on open-source software life cycles ^20^ was organized in July 2020. The objective of the workshop was to bring together the scientists’ communities and ESFRIs of astrophysics, astroparticle physics, and particle physics who are supporting, leading, or financing the software development within their domain. Common and best practices in software development were discussed and shared, enabling cross-fertilization across the domains. During the workshop a wide range of topics were presented and discussed, ranging from software engineering techniques through software licensing best practices to concrete examples of software to be onboarded. The outcomes of this workshop – *e.g.* recommendations on containerization and the need for a GitLab-Zenodo integration – were used to further shape the OSSR guidelines as well as the repository’s implementation. They form inputs for the ESCAPE Data Science schools for training young scientists to build and maintain the expertise in the field.

## 5 Adoption and interactions

The OSSR is more than a simple software catalog as it provides a place for researchers and developers of ESFRIs to collaborate, share knowledge, and access a wide variety of tools and resources. As such, it can be used by other services and projects to provide a common platform.

### 5.1 Integration with Matter and Technologies

Matter and Technologies (MT), a research program in the Helmholtz Association, combines development of both accelerators and detectors, and also focuses on the challenges of high-rate data ingestion from large-scale facilities, experiments, observatories, and accelerators. Data Management and Analysis (DMA) is one of three topics within MT, and focuses on and is driven by the data needs and challenges of the science within MT. The main goal of the Subtopic 2 of MT-DMA is to build an open-source repository with diverse software projects used for simulations and data analysis in Helmholtz institutes. Due to very similar objectives, requirements, and goals of OSSR and MT-DMA a decision towards a common effort was made and a cooperation was established. DMA software projects will be added to the OSSR repository as a sub-federation, profiting from well-established onboarding / curation processes, and metadata definition. In return, OSSR will gain more visibility and will be enriched by new entries from other fields such as gamma-and nuclear physics.

### 5.2 Integration with ESFRI Science Analysis Platform

The ESFRI Science Analysis Platform (ESAP) ^15, 16^ is a flexible toolkit designed to be used by ESCAPE project partners and others to easily deploy and customize their own platforms. The ESAP was created within a work package of the ESCAPE project and is able to connect together external services and other services developed by ESCAPE. To meet this goal, it is designed to be configurable, flexible, and extensible. This is achieved through the simplicity by which APIs can be integrated in the platform.

Services currently integrated into the ESAP include elements of International Virtual Observatory Alliance (IVOA) tools ^22^, various archives of ESFRIs and other observatories, a batch data processing framework and the possibility to search the ESCAPE data lake. For example, integrating IVOA tools gives the ESAP the ability to locate and access data for processing using Virtual Observatory (VO) systems, and use the IVOA-SAMP ^23^ standard.

A key feature of the ESAP is interactive analysis, which enables scientists to perform an analysis in real-time. Through the platform, users can search for and deploy software using JupyterLab ^24^ on binderhub-like ^21^ services ^25^. The list of available software includes eligible packages from the OSSR repository. The ESAP utilizes the eOSSR library (see Section 3.2) to search Zenodo for ESCAPE-community software. If the metadata of a software package contains the correct metadata entry, it will automatically be detected by the platform and be presented to users for deployment.

Additionally, from the ESAP-archive page, Zenodo can be accessed directly, also taking advantage of the eOSSR library. Users are able to search for entries of all types across all communities. The ESAP software stack was onboarded as an entry to the OSSR.

## 6 Conclusion and outlook

In conclusion, the ESCAPE Open-source Software and Service Repository (OSSR) serves as a central location for the dissemination and use of trusted open-source software in the fields of astronomy, astroparticle physics, and particle physics. Based on the general-purpose repository Zenodo, the OSSR is designed to make it easy for researchers, developers, and ESFRIs to find, access and download the software and services in their community, and to contribute their own tools and services. The curation process happening during the onboarding of software improves the quality of contributions, ensuring they comply to the FAIR principles, and makes the OSSR a source of trusted software that can be used for scientific analysis. The tools developed by the project, such as metadata generator, converter, and validator can ease the developers’ life by streamlining the software life cycle and could increase the number of contributions by lowering the barrier to sustainable open-science. The OSSR is also a place to share the know-how and best-practices of the community. By providing training on scientific software and analysis development and championing the FAIR principles, it accelerates their implementation within the community and ESFRIs. The OSSR will continue to grow and is supported by the ESCAPE Open Collaboration Agreement ^22^.

## Disclaimer

The views expressed in this article are those of the authors. Publication in Open Research Europe does not imply endorsement of the European Commission.

## Ethics and consent

Ethical approval and consent were not required

## Data Availability

No data are associated with this article

## Footnotes

^1^ https://about.zenodo.org/principles/

^2^ https://about.zenodo.org/terms/

^3^ https://home.cern

^4^ https://www.openaire.eu

^5^ https://purl.org/escape/ossr/eossr-documentation

^6^ https://about.gitlab.com/

^7^ https://docs.github.com/en/repositories/archiving-a-github-repository/referencing-and-citing-content

^8^ https://citation-file-format.github.io/

^9^ https://schema.org

^10^ https://github.com/inveniosoftware/invenio-swh/issues/4

^11^ https://CodeMeta.github.io/crosswalk/

^12^ https://projectescape.eu/

^13^ https://indico.in2p3.fr/category/841/

^14^ https://gitlab.in2p3.fr/escape2020/wp3

^15^ https://gitlab.com/escape-ossr/

^16^ https://www.asterics2020.eu/

^17^ https://slack.com/intl/en-gb/

^18^ https://www.youtube.com/live/TUrJ_IudVx8

^19^ https://github.com/escape2020/school2021, https://github.com/escape2020/school2022

^20^ https://escape2020.pages.in2p3.fr/wp3/wossl/

^21^ https://binderhub.readthedocs.io/en/latest/index.html

^22^ https://indico.in2p3.fr/event/27202/
