# Supplementary material for: The ESCAPE Open-source Software and Service Repository
Source: Open Res Eur. 2023 Nov 14;3:46. Originally published 2023 Mar 13. [Version 2] doi: 10.12688/openreseurope.15692.2 (PMC10804054; doi:10.12688/openreseurope.15692.2)
Supplement: Peer review history

# Reviewer report: Reviewer response for version 2

**DOI:** 10.21956/openreseurope.18144.r37054

**Published:** 2024-01-29

**Authors:** Kirstie J Whitaker

**Affiliations:** 1 Tools, Practices and Systems, The Alan Turing Institute (Ringgold ID: 522468), London, England, UK

I really appreciated reading this open letter. I found it a helpful overview of the ESCAPE open-source software and service repository.

 I want to particularly commend the ESCAPE team on describing the governance of the repository. I agree with them that what sets them apart from other similar resources is the fact there is a quality review step as part of archiving these software packages. Thank you for describing the curation process so clearly.

 I am reviewing the second version and feel that the authors have incorporated the recommendations from the reviewers of the first version well. In my opinion they have adequately addressed the concerns of the first reviewer.

Where applicable, are recommendations and next steps explained clearly for others to follow? (Please consider whether others in the research community would be able to implement guidelines or recommendations and/or constructively engage in the debate)

Yes

Does the article adequately reference differing views and opinions?

Yes

Are all factual statements correct, and are statements and arguments made adequately supported by citations?

Yes

Is the rationale for the Open Letter provided in sufficient detail? (Please consider whether existing challenges in the field are outlined clearly and whether the purpose of the letter is explained)

Yes

Is the Open Letter written in accessible language? (Please consider whether all subject-specific terms, concepts and abbreviations are explained)

Yes

Reviewer Expertise:

Open-source software, community management, data science and AI applied to scientific research, interoperability, data and software archiving, reproducibility. I'm also an expert in human neuroscience and have contributed to a data and software package standard for brain imaging data.

I confirm that I have read this submission and believe that I have an appropriate level of expertise to confirm that it is of an acceptable scientific standard.

# Reviewer report: Reviewer response for version 2

**DOI:** 10.21956/openreseurope.18144.r37040

**Published:** 2024-01-22

**Authors:** Cristóbal Bordiú

**Affiliations:** 1 Istituto Nazionale di Astrofisica, Bologna, Italy

This article nicely outlines the *Open Source Software and Service Repository* (OSSR) developed in the context of the ESCAPE project. Promoting Open Science practices, not only for data but also for software and services, is definitely crucial to maximise scientific exploitation and ensure reproducibility in this age of massive and increasingly complex datasets. Moreover, the emphasis on acknowledging developers’ effort and contribution is much needed. The significance of having such a software repository is thus evident.

 The article is well written and organised, providing sufficient context and a comprehensive overview of the project-specific goals before diving into the technical details of the OSSR implementation. In particular, the authors make a good effort in explaining the different assets that compose the OSSR from a high-level perspective. They also clearly underline what makes the OSSR different from other solutions like the ASCL.

 I only have a few minor comments/questions:

1. Sec. 1: " *…published,more…"* [missing whitespace]
2. Sec. 2.1: “... *suitable candidate contributions will be actively approached by the OSSR community*…” [How does the OSSR community monitor the “software ecosystem” to find those “suitable candidate contributions” in order to approach them?]
3. Sec 2.3: "... *the OSSR linksdevelopment platforms”...* [missing whitespace]
4. Sec 2.4: ". *..the relevance for the field of research serves as a selection criterion*” [how is the relevance evaluated? this may be a source of bias]
5. Sec 2.5: " *byreviewing*" [missing whitespace] // " *...Examples of reasons for removal… no new versions uploaded..." *[This could be dangerous. What period of time without new updates is needed to consider the software is no longer maintained? Anyway, well-built codes can still be useful even if they’re no longer maintained.]
6. Sec 3.1: " *FAIR4RS*" [expand the acronym on 1st appearance]
7. Sec 3.3: “ *The eOSSR uses Zenodo’s Application Programming Interface (API)* *to*…” [change to Zenodo's API, as API has been already expanded in Section 1]
8. Sec 3.4.2: " *even though (it is present) in the integration plan*"
9. Sec 3.5: " *...the process is reorganized on gitlab.com*” [it would be better to refer to Gitlab (the platform) instead of the webpage (gitlab.com), especially since you put the URL in the footnote.]

Where applicable, are recommendations and next steps explained clearly for others to follow? (Please consider whether others in the research community would be able to implement guidelines or recommendations and/or constructively engage in the debate)

Yes

Does the article adequately reference differing views and opinions?

Yes

Are all factual statements correct, and are statements and arguments made adequately supported by citations?

Yes

Is the rationale for the Open Letter provided in sufficient detail? (Please consider whether existing challenges in the field are outlined clearly and whether the purpose of the letter is explained)

Yes

Is the Open Letter written in accessible language? (Please consider whether all subject-specific terms, concepts and abbreviations are explained)

Yes

Reviewer Expertise:

Astrophysics & Software engineering

I confirm that I have read this submission and believe that I have an appropriate level of expertise to confirm that it is of an acceptable scientific standard.

# Reviewer report: Reviewer response for version 1

**DOI:** 10.21956/openreseurope.16954.r33278

**Published:** 2023-07-19

**Authors:** Diomidis Spinellis

**Affiliations:** 1 Athens University of Economics and Business, Athens, Greece; 2 Department of Software Technology, Delft University of Technology, Delft, South Holland, The Netherlands

This paper describes the goals and high-level implementation of a repository to host software in the fields of astronomy, astroparticle physics, and particle physics. This is interesting work, which however requires many improvements and considerable refinement.

 Regarding soundness, the study's contributions seem to address its goals, but in some cases the applied research methods lack the required rigor. It is unclear what specific requirements drove the development of the platform and how these were collected. At no point does the text clarify how services (as mentioned in the title) are supported by the repository. The library's API is neither described nor referenced. Importantly no attempt is made to evaluate the platform. Threats to validity and their effect on the results' soundness are not explicitly presented.

 Regarding significance, no motivation is provided for performing the research. The work's potential impact to the field is clear, but not substantiated.

 Regarding novelty, there is no systematic presentation of existing work in the area. It is not apparent how the work's contributions offer an improvement over to the state-of-the-art. Many other repositories that can, in theory, satisfy the described goals exist. No empirical evidence is offered to show that ESCAPE improves upon the state of the art. A table listing similar repositories and the quantitative and qualitative attributes of each one would help in this direction.

 Regarding verifiability and transparency, the method's description and the provided data allow the independent verification of the outlined findings. The study's verifiability would benefit by providing additional materials to support independent verification or replication of the paper’s claimed contributions. In particular the study would benefit from a quantitative analysis of the repository's and the library's usage in terms of uploads, downloads, citations, and content and the extent to which the stated goals have been satisfied.

 Regarding presentation, the paper's structure can be improved. In a number of places (see below) terms are used before they are introduced. The paper does not follow a typical research study structure. This would not be a problem, if the corresponding material (methods, results, related work) was present and easily identifiable, which however is not the case. The writing is clear and easy to understand. The paper is mostly well-written, though some typos and grammar errors can distract the reader. Careful copy-editing is required. Some minor issues are identified below. The formatting follows the provided instructions. Note that superscripts are commonly set after commas and periods. The abstract is self-standing and summarizes succinctly the paper's essence. The paper could benefit from the addition of figures in particular ones visualizing quantitative attributes of the repository's use.

**Minor comments**

- Section -1: Please start with a short description of the problem and current solutions, followed by your contribution. The project can be described later on as an enabler for your work or part of your methods.
- Section -Summary: Consider changing "is reliable and open-source" into "is reliable and distributed under an open-source license".
- Please expand  all initials FAIR ,SIRS, WOSSL)  on their first use
- Section -2.1: Please rephrase the content to reflect the research's goals rather than outcomes.
- Section -2.2: Please start by introducing the FAIR principles and their importance to scientific research
- Section -2.3: Consider changing "such that" into "so that".
- Section -2.3: Consider changing "trustability" into "trustworthiness".
- Section -2.4: Please specify, how often does the board meet? How is the board governed? How are outdated entries detected and reviewed?
- Section -3.2: Please specify which specific policies are validated through the library. What prevents a cloned library that skips validation to push a contribution to OSSR?
- Section -3.3: Consider changing "docker" into "Docker".
- Section -3.4: Please introduce CodeMeta before referring to it.
- Section -3.4.3: Please clarify how these tools are used. Are they accessed through the command line, do they offer a graphical interface?

Where applicable, are recommendations and next steps explained clearly for others to follow? (Please consider whether others in the research community would be able to implement guidelines or recommendations and/or constructively engage in the debate)

Partly

Does the article adequately reference differing views and opinions?

No

Are all factual statements correct, and are statements and arguments made adequately supported by citations?

Partly

Is the rationale for the Open Letter provided in sufficient detail? (Please consider whether existing challenges in the field are outlined clearly and whether the purpose of the letter is explained)

No

Is the Open Letter written in accessible language? (Please consider whether all subject-specific terms, concepts and abbreviations are explained)

Partly

Reviewer Expertise:

Software engineering

I confirm that I have read this submission and believe that I have an appropriate level of expertise to state that I do not consider it to be of an acceptable scientific standard, for reasons outlined above.

# Response

**Published:** 2023-10-27

**Authors:** Thomas Vuillaume

**Affiliations:** LAPP, CNRS, France

We thank the reviewer for their time and effort in reviewing our work. Their comments and feedback were essential to improve the paper and we have taken them into consideration to better explain the genesis and goals of the ESCAPE OSSR. Upon careful consideration of their comments, we recognize that there appears to be a difference in perception regarding the scope and purpose of the article, as well as the thematic focus of the journal to which we are submitting. Specifically, it seems that the main point of contention is whether the paper is intended to function as an overview or a technical paper. As an open letter, our intent here is not to be technically exhaustive but to present the views and ideas behind the ESCAPE OSSR. Previous works, cited here, present these technical details more thoroughly. We have revised the manuscript to better reflect its purpose. In addition, the ESCAPE project deliverables ( https://projectescape.eu/deliverables-and-reports) deliver a more in-depth technical view of the OSSR, in particular https://projectescape.eu/deliverables-and-reports/d33-conceptual-design-report-software-and-service-repository and https://projectescape.eu/deliverables-and-reports/milestone-17-status-common-software-and-services-proposal-and-integration. All the minor comments have been corrected in the second version of the manuscript and we would like to provide some answers to your valid questions: Regarding the API, it appears that there might be a misunderstanding of its goal as it does not provide an interface to the hosted services but to the OSSR itself, allowing automated management of its content. The technical description of the API is done in 10.48550/arXiv.2212.00499 and in the online, up-to-date, documentation (https://escape-ossr.gitlab.io/eossr/), we believe it was not the purpose of this paper to present it. The services are not provided through the OSSR, they are recorded via their software or documentation in the OSSR. The OSSR API is therefore not designed to access these services directly.

# Reviewer report: Reviewer response for version 1

**DOI:** 10.21956/openreseurope.16954.r31770

**Published:** 2023-07-12

**Authors:** Michael Zingale

**Affiliations:** 1 Department of Physics and Astronomy, Stony Brook University, Stony Brook, New York, USA

This article describes an open source software repository, ESCAPE. This is a very nice project and the authors are clearly excited about promoting open source scientific software. Overall, I only have minor comments.

 There are a lot of acronyms in the article, which makes it very difficult to read. For example FAIR is used 4 times before it is actually defined. SIRS is never defined. These acronyms should be defined when they are first used. Even better would be to include a table of abbreviations.

 It is a little unclear what type of software is welcomed in the ESCAPE repository. The authors should list some of the packages (and their uses) that are already in ESCAPE. My read of this article suggests that ESCAPE is primarily for experimental/observational codes, but what about simulation/theoretical codes?

 More discussion about the relation to other repositories would be useful. For example, how does this differ from the goals of the Astronomy Source Code Library (ASCL)?

 The procedure by which a package is added to ESCAPE should be expanded. Can the author of a package ask to be included? And what if ESCAPE wants to add a package but the authors of that package don’t want to be included – is there a procedure for that?

 Overall this is a nice resource, and my comments above are primarily meant to help readers more clearly understand the goal and how they will benefit from ESCAPE.

Where applicable, are recommendations and next steps explained clearly for others to follow? (Please consider whether others in the research community would be able to implement guidelines or recommendations and/or constructively engage in the debate)

Partly

Does the article adequately reference differing views and opinions?

Yes

Are all factual statements correct, and are statements and arguments made adequately supported by citations?

Yes

Is the rationale for the Open Letter provided in sufficient detail? (Please consider whether existing challenges in the field are outlined clearly and whether the purpose of the letter is explained)

Yes

Is the Open Letter written in accessible language? (Please consider whether all subject-specific terms, concepts and abbreviations are explained)

Yes

Reviewer Expertise:

Computational astrophysics

I confirm that I have read this submission and believe that I have an appropriate level of expertise to confirm that it is of an acceptable scientific standard.

# Response

**Published:** 2023-10-27

**Authors:** Thomas Vuillaume

**Affiliations:** LAPP, CNRS, France

We thank the referee for their time and effort reviewing our work. We believe all comments have been addressed in the version 2, in particular:

- the missing acronyms definitions have been added;
- a section comparing the OSSR to similar initiatives, especially the ASCL, has been added (section 2.6);
- The OSSR is open to all community software and code bases, observational and theoretical, there are already entries of both in the repository. Wwe added this OSSR aim to the text (section 2.1) as well as a the link to the always up-to-date list of entries (section 5.1);
- Software is added by the authors of the code themselves, it is not pulled by the ESCAPE community. This has been made clearer in the text (section 2.1) now.
